# Protein–Protein Interactions in Base Excision Repair

**DOI:** 10.3390/biom15060890

**Published:** 2025-06-18

**Authors:** Govardhan Rathnaiah, Joann B. Sweasy

**Affiliations:** Fred and Pamela Buffett Cancer Center and Eppley Institute for Cancer Research, Omaha, NE 68198, USA; grathnaiah@unmc.edu

**Keywords:** DNA glycosylase, poly (ADP-ribose) polymerases, XRCC1, APE1, DNA polymerase, ligase

## Abstract

The Base Excision Repair (BER) pathway involves a highly coordinated series of protein–protein interactions that facilitate the recognition, excision, and repair of damaged bases. Key enzymes such as DNA glycosylases, apurinic/apyrimidinic endonuclease 1 (APE1), polynucleotide kinase-phosphatase (PNKP), DNA polymerase b (Pol β), ligase IIIα (LigIIIα), poly (ADP-ribose) polymerases PARP1 and PARP2, and X-ray repair cross-complementing protein 1 (XRCC1) catalyze BER in a tightly regulated molecular network. These interactions ensure the seamless handoff of DNA intermediates between the core enzymes of the BER pathway. Understanding the details of protein–protein interactions in BER provides valuable insights into the molecular underpinnings of DNA repair processes. In this review, we focus on protein–protein interactions between the components of the single-nucleotide BER (SN-BER) pathway and other proteins that interact with BER components and regulate the coordination of the pathway. We also briefly discuss the interactions of other proteins that interact with the components of SN-BER based on functional evidence.

## 1. Introduction

Base excision repair (BER) is a crucial cellular mechanism responsible for processing DNA damage, including single-base modifications and abasic sites, which can arise from oxidative stress, alkylation, deamination, and spontaneous hydrolysis [1]. The efficiency and accuracy of BER are paramount for maintaining genomic stability and preventing mutations that could lead to cancer [2]. Central to the BER pathway are a series of coordinated protein–protein interactions that facilitate the recognition, excision, and repair of damaged bases [3,4,5]. These interactions ensure the seamless handoff of DNA intermediates between the core enzymes of the BER pathway, such as DNA glycosylases, apurinic/apyrimidinic endonuclease 1 (APE1), polynucleotide kinase-phosphatase (PNKP), DNA polymerases (Pol β), and ligase IIIα (LigIIIα) [6]. Additional proteins such as poly (ADP-ribose) polymerases, PARP1 and PARP2 (PARP), and X-ray repair cross-complementing protein 1 (XRCC1) play important roles in facilitating interactions between the core enzymes. Understanding the dynamics and regulation of these protein–protein interactions provides valuable insights into the molecular underpinnings of DNA repair processes and highlights potential therapeutic targets for enhancing DNA repair in disease contexts.

Coordination of BER is crucial for efficient repair of base lesions and for minimizing the effects of toxic repair intermediates. Two models have been proposed for coordinated repair. The first, called “Passing the Baton”, is a model that involves transient interactions between proteins to efficiently hand off intermediates from one enzyme to the next in the pathway [4,7,8]. This model invokes an underlying mechanism of substrate channeling during BER and is based on in vitro analyses of BER with purified enzymes. Evidence for substrate channeling was provided by demonstrating that the APE1 protein retains the cleaved DNA intermediate and likely transfers this intermediate to Pol b. The second model, called the “BERosome Repair Complex”, is a model invoking the existence of a preexisting stable repair complex [9,10]. For example, the NEIL 1 DNA glycosylase is present in large protein complexes that contain the replication machinery. The findings indicate that the C-terminal domain of DNA glycosylase NEIL1 interacts with DNA replication proteins, such as proliferating nuclear antigen (PCNA), replication factor C, Pol δ, and DNA Ligase (Lig I). Disruption of this complex inhibits BER completion and chromatin localization. Post-translational modifications (PTMs) of BER proteins are likely to play a role in their interactions that are important for processing DNA damage (for a review, see [11]). Studying these interactions will help establish how the overall pathway is coordinated during the repair of DNA damage. In this review, we will focus on protein–protein interactions between the components of the single-nucleotide BER (SN-BER) pathway. Readers are encouraged to refer to the recent review by Moor et al. [12] for detailed information on the proteins involved in other sub-pathways of the BER.

## 2. Single Nucleotide BER (SN-BER) Pathway

**Figure 1:**
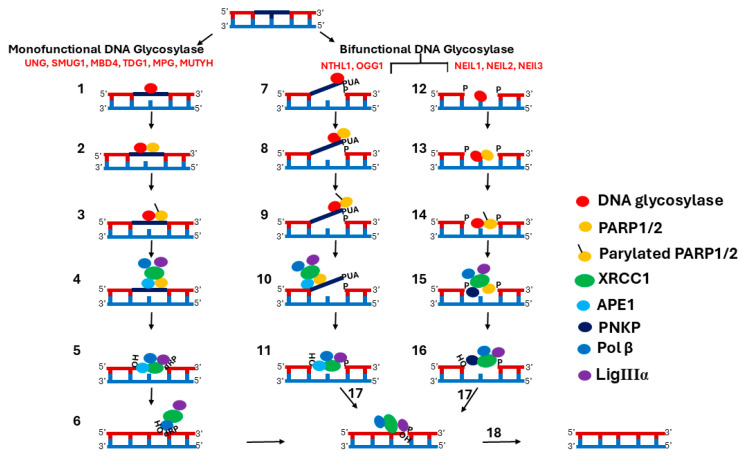
Scheme of Multi-Step BER. (**1**) Monofunctional DNA glycosylases remove the base lesion by cleaving the N-glycosidic bond between the damaged base and the sugar phosphate backbone of the DNA, creating an AP site. (**2**) PARP recognizes and binds to AP sites, resulting in stimulation of their activity. (**3**) Activated PARP synthesizes PAR on itself and displaces DNA glycosylases. (**4**) XRCC1 binds to PAR on the PARP and is recruited to the damage site. APE1, Pol β, and LigIIIα are also recruited, along with XRCC1. (**5**) APE1 cleaves the AP site, creating an SSB with a 1-nucleotide gap and 3′-OH and 5′-dRP termini. (**6**) Pol β fills the gap with the correct nucleotide and removes the 5′-dRP, leaving a 5′-P. (**7**) Bifunctional glycosylases such as NTHL1 and OGG1, using their glycosylase activity, remove the damaged base by cleaving the N-glycosidic bond, generating an AP-site, and then with their AP lyase activity, incise the AP-site on the 3′-side, creating an SSB with 3′-α, β unsaturated aldehyde (3″-PUA), and 5′- P termini. (**8**) PARP recognizes and binds to SSB, resulting in stimulation of its activity. (**9**) Activated PARP synthesizes PAR on itself and displaces DNA glycosylases. (**10**) XRCC1 binds to PAR on the PARP and is recruited to the damage site. APE1, Pol β, and LigIIIα are also recruited, along with XRCC1. (**11**) The 3′ to 5′ exonuclease activity of APE1 removes PUA at the 3′ end and adds an OH. (**12**) Bifunctional glycosylases, such as NEIL1, 2, and 3, with their glycosylase activity, remove the damaged base by cleaving the N-glycosidic bond, generating the AP site, and then, with their AP lyase activity, incise the AP-site on the 3′-side, creating 3′-P and 5′-P ends. (**13**) PARP recognizes and binds to the SSB, resulting in stimulation of its activity. (**14**) Activated PARP synthesizes PAR on itself and displaces DNA glycosylases. (**15**) XRCC1 binds to PAR on PARP and is recruited to the damage site. PNKP, Pol β, and LigIIIα are also recruited, along with XRCC1. (**16**) The 3′ phosphatase activity of PNK removes 3′ P and adds OH. (**17**) The polymerase activity of Pol β fills the gap by adding the correct nucleotide. (**18**) LigIIIα seals the nick. Note: PARP1/2 involvement in the SN-BER pathway is not mandatory as it depends on the damaged substrate and the type of DNA glycosylase that repairs the damage.

The SN-BER pathway is initiated by different lesion-selective DNA glycosylases with overlapping substrate specificity, resulting in the removal of the base lesion and generation of AP sites and single-strand breaks (SSBs) [13,14]. The steps involved in the SN-BER pathway are depicted in Figure 1. SN-BER initiates with the recognition and removal of a base lesion by a DNA glycosylase, which generally recognizes the base damage through a process of DNA scanning that is not completely understood [15]. There are two main types of DNA glycosylases: monofunctional and bifunctional DNA glycosylases [16]. In mammals, six monofunctional DNA glycosylases have been identified: uracil DNA glycosylases (UNG) [17,18,19], single-strand-specific monofunctional uracil DNA glycosylase (SMUG1) [20,21], methyl-CpG binding domain protein 4 (MBD4 or MED1) [22,23], thymine DNA glycosylase (TDG) [24,25], alkyladenine DNA glycosylase (AAG or MPG) [26], and MutY homolog (MUTYH) [27]. Monofunctional DNA glycosylases remove the base lesion by cleaving the N-glycosidic bond between the damaged base and the sugar phosphate backbone of the DNA, creating apurinic/apyrimidinic (AP) sites [18]. There are five bifunctional DNA glycosylases: Nth-like DNA glycosylase 1 (NTHL1) [28], 8-oxoguanine DNA glycosylase (OGG1) [29], endonuclease VIII-like 1 (NEIL1), NEIL2, and NEIL3 [30,31,32]. In addition to glycosylase activity, bifunctional DNA glycosylases possess AP lyase activity that cuts the phosphodiester bond of DNA, creating an SSB. Bifunctional DNA glycosylases are subdivided into two groups based on the AP lyase reaction mechanism [33]. One mechanism is the β-elimination reaction. Both NTHL1 and OGG1 incise the AP-site on the 3′-side, creating an SSB with 3′-α, β unsaturated aldehyde (3′-PUA) and 5′-P termini [34,35]. A second lyase mechanism is the β/δ-elimination reaction. The NEIL1, 2, and 3 DNA glycosylases incise the AP-site on the 3′-side, creating 3′-P and 5‘-P ends [36]. However, the AP lyase activity of bifunctional DNA glycosylase is very slow, so it is often bypassed in the presence of APE1 [37,38,39]. A study on bifunctional DNA glycosylase NTHL1 reported that AP lyase function is dispensable for the BER of naked DNA, but it is important for the BER in nucleosomes [39].

Next, AP sites and SSBs are recognized and bound by PARP1 and PARP2 (PARP) [40] (Figure 1). PARP comprises key regulatory proteins stimulated by DNA interruptions [41]. Two types of PARP, PARP1 and PARP2, play an important role in the repair of SSBs as part of the BER pathway [42]. For more detailed information on the involvement of PARP1 and PARP2 in BER, please refer to the recent reviews by Zhang et al. and Lavrik et al. [43,44]. PARP1 and PARP2 have redundant functions in BER, and the disruption of either protein alone does not significantly impact BER [45]. However, it was demonstrated that the simultaneous disruption of both proteins leads to an increase in DNA strand breaks. The C-terminus of PARP1 and PARP2 is conserved and contains a catalytic domain (CAT) and a TRP-Gly-Arg (WGR) domain. But the N-terminal region (NTR) is not conserved [46,47,48]. The PARP1 N-terminal DNA-binding domain (DBD) is longer and contains three Zn finger domains and a BRCT domain, while PARP2 has a shorter N-terminal DBD [47]. The DBD domain of PARP1 binds to AP sites and SSBs, resulting in stimulation of its poly ADP-ribosylation (PARylation) activity [49,50]. Activated PARP1 synthesizes poly (ADP-ribose) (PAR) on itself and chromatin-associated proteins [51,52]. The accumulation of PAR chains on chromatin-associated proteins results in chromatin reorganization, providing access to AP sites and SSBs for downstream BER components [53].

XRCC1 binds to the PAR chains on PARP1 and is recruited to the BER repair site [54]. Auto-PARylation of PARP1 inhibits its enzyme activity and decreases its affinity for SSBs, leading to the release of PARP1 from the repair site [55,56]. The interaction between XRCC1 and PARP1 is crucial for the assembly of multi-protein complexes at the repair site [57]. PARP1 and XRCC1 play a central coordinating role by linking the upstream and downstream BER components for efficient transfer of repair intermediates from one enzyme to another [52,53]. XRCC1 interacts with multiple BER components, including DNA glycosylases such as UNG2, OGG1, NTHL1, MPG, NEIL1, NEIL2, PARP1, APE1, PNKP, Pol β, and LigIIIα [58,59,60,61] (Figure 1). XRCC1 has three domains: the XRCC1 N-terminal domain (X1NTD), which interacts with a gapped DNA-Pol β complex [62]; the central BRCT domain (X1BRCTa) that binds to PAR on PARP1 [54]; and the C-terminal BRCT domain (X1BRCTb) that interacts with LigIIIα [63]. Acting as a scaffolding protein, XRCC1 stabilizes APE1, PNKP, DNA Pol β, and LigIIIα at the repair site [64,65,66,67,68].

AP sites generated by monofunctional DNA glycosylases are handed off to APE1, which cleaves the DNA backbone, creating a single-nucleotide gap that includes 3′-OH and 5′-dRP termini (Figure 1). Next, Pol β fills the gap with the correct nucleotide and removes the 5′-dRP, leaving a 5′-P. SSBs generated by bifunctional DNA glycosylases with the β-elimination reaction mechanism are further processed by APE1. The 3′ to 5′ exonuclease activity of APE1 removes PUA at the 3′ end and generates a 3′-OH to prepare the ends for gap filling by Pol β [61,69]. SSBs generated by a bifunctional DNA glycosylase with a β/δ-elimination reaction mechanism are handed off to PNKP. The DNA 3′ phosphatase activity of PNKP processes the 3′-P, resulting in the generation of a 3′-OH [61]. Next, Pol β fills the gap by adding a single nucleotide [70]. Finally, LigIIIα seals the nick. Some AP sites generated during the BER repair process have dirty breaks, and LigIIIα processes that dirty break, resulting in abortive ligation intermediates [71]. These have an adenylate group at the 5′-P nicks [72]. Aprataxin’s (APTX) proofreading function removes the adenylate group from the 5′ nicks to reveal the 5′-P termini, permitting continuation of the repair process [73].

## 3. Interaction Between SN-BER Components

### 3.1. DNA Glycosylase—PARP1 Interaction

DNA glycosylases, such as OGG1 and NEIL1, have been shown to bind to and stimulate PARP1 [74,75]. The N-terminus of OGG1 and the C-terminus of NEIL 1 interact with the BRCA1 C-terminal (BRCT) domain of PARP1 [74,75]. This binding is enhanced in the presence of oxidative stress [75]. OGG1 and NEIL1 DNA glycosylases stimulate PARP1, which inhibits them and leads to the release of the DNA glycosylase from the damaged site, allowing downstream repair components to engage [2,74,75]. PARP1 has also been shown to add PAR on the N-terminal and CORE domains of TDG, enhancing its enzymatic turnover by promoting its dissociation from the AP sites [41]. DNA glycosylases bind to their product with high affinity, which is a rate-limiting step in the completion of repair [76]. PARylation of TDG facilitates its release from the AP site, thereby promoting efficient repair. PARP1 thus regulates the activity of DNA glycosylases, ensuring the repair process is tightly controlled and coordinated with downstream components of the BER pathway. However, besides OGG1, NEIL1, and TDG, interactions of PARP1 with other DNA glycosylases are not well studied.

### 3.2. Interaction of DNA Glycosylases with Downstream BER Components

DNA glycosylases bind tightly to AP sites and SSBs, which leads to product inhibition. This tight binding is beneficial as it helps sequester the potentially toxic intermediate product and efficiently passes it to APE1 [76]. Several studies have shown that APE1 stimulates the turnover of TDG, MUTYH, and OGG1 DNA glycosylases by accelerating the rate-limiting product release step [76,77,78,79,80,81,82,83,84]. Two primary mechanisms have been proposed for the stimulation of DNA glycosylase turnover by APE1. One is an active mechanism in that APE1 physically interacts with the glycosylase, facilitating its release from the AP site and allowing the repair process to proceed. This has been demonstrated for TDG [76,80] and MUTYH [82]. The second one is a passive mechanism, as there is no physical interaction between the glycosylase and APE1. Instead, APE1 may induce conformational changes in the DNA or the glycosylase itself, promoting the release of the glycosylase from the AP site without direct contact [78,79]. The passive mechanism by which APE1 stimulates the DNA glycosylase involves the kinetic trapping mechanism and has been demonstrated for OGG1. The authors propose that “dynamic excursions” of OGG1 from the AP site allow APE1 to invade the site and rapidly incise the phosphate backbone [79]. This mechanism entails the kinetic trapping of the AP site through the pre-association of APE1 with DNA dynamic excursions. Further details on kinetic trapping are beyond the scope of this review, and readers are encouraged to refer to the original publication for more information.

DNA glycosylases, such as UNG2, OGG1, NTHL1, MPG, NEIL1, and NEIL2, and APE1 interact with XRCC1 [58,59,60,61]. OGG1, NTHL1, MPG, and APE1 have been shown to interact with the same region of XRCC1, the hinge region between the X1NTD and X1BRCT1 domains [59,60]. UNG2 interacts with the nuclear localization signal region (NLS) of XRCC1 [58]. These studies demonstrated that XRCC1 stimulates DNA glycosylase activity by stabilizing the DNA glycosylase at the repair site. One study observed that XRCC1 and APE1 have an additive effect on OGG1 activity [60]. Considering that both DNA glycosylases and APE1 interact with the same region of XRCC1, these studies suggest that when APE1 binds to XRCC1, it displaces the DNA glycosylase and results in the handoff of the repair site to APE1.

DNA glycosylases of the NEIL family initiate the APE1-independent SN-BER pathway and pass the repair intermediate to PNKP for further processing [61,85]. XRCC1 has been shown to interact with NEIL1 and NEIL2 [59,61]. These studies reported that NEIL1 and NEIL2 interact with XRCC1, Pol β, and LigIIIα, but not with PNKP [61,85]. However, PNKP has been shown to interact with XRCC1 and LigIIIα in a different study [67]. NEIL1 and NEIL2 interact with the N-terminus of Pol β and the C-terminus of LigIIIα [61,85]. It is suggested that although PNKP does not interact directly with NEIL1, it is associated in a larger complex with XRCC1, Pol β, and LigIIIα [67]. Another study reported that the C-terminal common interaction domain (CID) of NEIL1 plays a critical role in the coordination of the repair process by interacting with several downstream BER components [86]. This study reported that the CID domain of NEIL1 is dispensable for glycosylase activity but important for efficient repair of oxidized DNA bases. The results revealed that the NEIL1 CID domain interacts with PNKP, XRCC1, Pol β, and LigIIIα. These studies indicate that XRCC1 forms a multiprotein complex with NEIL1, PNKP, Pol β, and LigIIIα. This complex is critical for the efficient repair of oxidized DNA bases in the APE1-independent SN-BER pathway [67,86].

DNA glycosylases AAG/MPG and OGG1 have been shown to interact with Pol β and DNA polymerase λ (Pol λ) [87]. Specifically, co-immunoprecipitation (Co-IP) experiments demonstrated protein–protein interactions and suggested that these interactions may be important in coordinating the activities of Pol β and Pol λ in BER. Another study demonstrated that AAG/MPG, OGG1, NTHL1, MBD4, UNG2, and SMUG1 stimulate the Pol β polymerase activity [88], suggesting that DNA glycosylases can recruit Pol β to the BER repair site and form a multiprotein complex.

### 3.3. Interaction Between Downstream Components of SN-BER

XRCC1 interacts with the N-terminus of APE1, stimulating its enzymatic activity [89]. Genotoxic stress and the deacetylase activity of SIRTUIN1 (SIRT1) have been shown to promote an interaction between APE1 and XRCC1 [68]. Additionally, PARP1 increases the rate of strand incision activity of APE1, indicating a functional partnership between PARP1 and APE1 in the BER pathway [90]. APE1 exhibits high affinity with its product, causing it to remain bound to the incised AP site. PARP1 also binds to the incised AP site. While both APE1 and PARP1 bind to the same BER intermediates, the details of this interaction are not fully elucidated. A recent study has shown that covalent modification of APE1 by PARylation results in dissociation of APE1 from the incised AP site, making way for the downstream BER enzymes to continue the repair process and stimulate enzymatic turnover of APE1 [41].

In addition to AP endonuclease activity, APE1 also possesses 3′ to 5′ exonuclease activity [91]. It has been suggested that APE1 exonuclease may play a proofreading role to remove the mismatched bases added by the relatively low-fidelity DNA polymerase Pol β. Another study demonstrated, with high-resolution APE1-DNA structural studies, that APE1 removes 3′ mismatches at a DNA nick and regenerates a 1-nt gapped DNA product [92]. The APE1-generated SSB with a 1-nucleotide gap is a substrate for Pol β, and it has been suggested that the APE1 product is channeled to Pol β as a part of the “passing the baton” model [4,93]. A recent study has supported the combination of “passing the baton” and “BERosome Repair Complex” models for the coordination of downstream steps in the BER pathway [94]. Specifically, it was suggested that Pol β can displace APE1 from the damaged DNA site, and that it forms a ternary complex with APE1 and the DNA intermediate. Using fluorescence resonance energy transfer (FRET) and total internal reflection fluorescence microscopy, other studies have demonstrated that Pol β binds to APE1 and the incised AP site, forming a transient ternary complex [95,96]. In most of the observations, Pol β dissociated from the ternary complex shortly after binding, but, in some cases, the binding of Pol β resulted in the dissociation of APE1 from the ternary complex. It was suggested that the transfer of the incised AP site from APE1 to Pol β is dependent on the dissociation kinetics of APE1.

The C-terminal domain of Pol β interacts with the X1NTD domain of XRCC1 [66]. The XRCC1 amino acid residue V86 has been shown to be important for interaction with Pol β [97]. This study reported that the V86R mutation of XRCC1 resulted in complete loss of interaction with Pol β. Previously, the interaction of XRCC1 with Pol β was thought to be important to prevent the degradation of Pol β [98]. However, a recent study demonstrated that this interaction is very important for the recruitment of Pol β to the BER repair site [65]. The data suggest that evolutionarily conserved Pol β amino acid residues L301 and V303 are crucial for the interaction with XRCC1. Disruption of either residue alone resulted in significantly reduced recruitment of Pol β to the repair site. Simultaneous disruption of both residues resulted in a loss of interaction with XRCC1 and no detectable Pol β at the repair site.

The X1BRCTb domain of XRCC1 interacts with the C-terminal domain of DNA LigIIIα [99]. The amino acid residues 573–592 in the X1BRCTb domain are critical for the interaction of XRCC1 with LigIIIα [100]. A 20-mer peptide, comprising residues 573–592 in the C-block motif of the X1BRCTb domain, is sufficient to interact with the LigIIIα. A separate study demonstrated that Trp74 in the X1BRCTb domain is critical for the interaction with LigIIIα [101]. The C-terminal 149 amino acids of LigIIIα are sufficient to interact with XRCC1. XRCC1 has been shown to interact with APTX [102,103]. These studies reported that APTX plays a role in maintaining the steady-state level of XRCC1. They demonstrated that casein kinase 2 (CK2) phosphorylation of XRCC1 at amino acid residues S518, T519, and T523 regulates its binding to the FHA domain of APTX. For more detailed information on the structural domains of XRCC1 interacting with Pol β, LigIIIα, and APTX, please refer to the review by London et al. [63].

LigIIIα has been shown to directly interact with tyrosyl DNA phosphodiesterase 1 (TDP1) [104]. The TDP1 catalytic domain interacts with the DNA-binding domain of LigIIIα. It was shown that TDP1 forms a complex with LigIIIα alone or the XRCC1-LigIIIα complex. TDP1 is involved in the removal of 3′-phosphotyrosine adducts generated by proteolytic degradation of covalent topoisomerase 1(TOP1)-DNA complexes. The interaction between TDP1 and LigIIIα is likely important for the recruitment of XRCC1 and partner proteins to repair the SSB. The S81 residue in the N-terminal domain of TDP1 is important for interactions with LigIIIα [105]. The findings indicate that the mutation of S81 to alanine resulted in the loss of interaction with LigIIIα.

Studies using fluorescence-based techniques and light scattering-based techniques demonstrated multiple functional interactions between downstream components of BER [96,106], including the ternary complex of XRCC1-Pol b- LigIIIα. These studies also demonstrated direct interactions between APE1-Pol β, APE1-PARP1, APE1-TDP1, and Pol β-TDP1. A recent study has shown functional coordination between APE1, Pol β, and LigIIIα/ LigI in the downstream steps of the BER pathway [64]. This study focused on protein interaction kinetics using a surface plasmon resonance assay in real time. The findings indicate that both APE1 and Pol β interact with the catalytic core/BRCT domain of LigIIIα. They also observed that both Pol β and LigIIIα interact with XRCC1 and PARP1. Furthermore, they validated the tight interactions between Pol β and APE1 with LigIIIα and XRCC1 using size-exclusion chromatography (SEC) and provided evidence for the BER multiprotein complex consisting of XRCC1, Pol β, and LigIIIα. Using a biolayer interferometry assay, it was demonstrated in real time that Pol β and LigIIIα bind to a one-nucleotide gap DNA efficiently. The authors reported K_D_ values for the protein complexes of 7 nM for LigIIIα and 20 nM for Pol β.

## 4. Interaction of BER Components with Other Proteins

The BioGRID database (https://thebiogrid.org/) (8 November 2024) was used to create the protein interactome network of SN-BER components. BioGRID is a biomedical interaction repository with data compiled through the comprehensive curation of published protein–protein interaction studies. The protein–protein interaction networks that were generated contain interactions identified by both high-throughput and low-throughput studies (Figures S1–S16). In this review, we will briefly discuss the proteins for which evidence for direct interaction with BER components is provided by low-throughput studies (Table 1), such as Co-IP, yeast two-hybrid assays, and pull-down assays, as these studies provide functional evidence for protein interactions.

### 4.1. Uracil DNA Glycosylases

The N-terminal region of UNG2 interacts with the C-terminal region of replication protein A (RPA2) [107]. It was suggested that RPA2 may recruit UNG2 to the DNA replication complex for the purposes of scanning for uracil. Another study by the same group reported that UNG2 binds to RPA and PCNA and co-localizes at the replication fork [108]. The results revealed that the N-terminus of UNG2 interacts with PCNA. These studies indicate that the UNG2-mediated BER pathway takes place in replication foci where UNG2 co-localizes with RPA and PCNA.

The CRL4 E3 ubiquitin ligase complex has been shown to regulate the steady-state levels of UNG2 and SMUG1 [109]. UNG2 physically interacts with the DDB1 and CUL4-associated factor 1 (DCAF1) component of the CRL4 complex. The human immunodeficiency virus type 1 (HIV-1) Vpr protein has also been shown to bind to UNG2 and SMUG1 and enhance their interaction with DCAF1, leading to increased degradation of UNG and SMUG1 by CRL4-mediated ubiquitination [110]. Another study reported that Vpr loads the UNG2 onto the DCAF1 component of the CRL4 complex, leading to degradation of UNG2 [111]. This has been proposed to lead to an alteration of the humoral B-cell response in patients infected with HIV [112].

### 4.2. MBD4

MBD4 has been shown to interact with the MLH1 mismatch repair (MMR) complex [23]. Another study reported that MBD4 and MLH1 were found in a complex with the Fas-associated death domain protein (FADD) [113]. FADD is a key adaptor protein involved in apoptosis. The interaction between FADD, MBD4, and MLH1 is suggested to play a role in regulating the apoptotic response to diverse DNA lesions. MBD4 also plays a role in epigenetic silencing (transcriptional repression) in cancer by interacting with histone deacetylase (HDAC), switch-insensitive (Sin3A), and repressive polycomb proteins [114,115]. MBD4 has been implicated in regulating the activity of DNA maintenance methyltransferases (DNMT1) by interacting with the UHRF1 E3 ubiquitin ligase and USP7 [116]. MBD4 is also regulated by SUMOylation [117]. Specifically, the data suggest that small ubiquitin-like molecule 1 (SUMO1) SUMOylates MBD4 at K137, K215, and K377, indicating that MBD4 is SUMOylated in a DNA damage-specific manner.

### 4.3. TDG

TDG interacts with transcriptional coactivators CREB binding protein (CBP) and p300 [118], and a previous study suggested that CBP/p300 regulates TDG by acetylation. TDG acetylation regulates the recruitment of APE1 to the AP site. A separate study demonstrated that TDG interacts with the XPC-HR23B complex, and that this interaction stimulates the turnover of TDG in the presence of APE1 [119].

DNA methyltransferases DNMT3a and DNMT3b have been shown to interact and potentiate mismatch repair by TDG [120,121]. TDG binds to DNMT3a and promotes the degradation of DNTM3a by ubiquitination [121]. DNMT3b interacts with TDG and colocalizes with the heterochromatin. It appears that the majority of T:G mismatches are localized to the heterochromatin, and that the interaction of TDG either with DNMT3b or DNTM3a could serve to recruit TDG to the mismatch repair site. Ring finger protein 4 (RNF4) has been shown to interact with and enhance the enzymatic activity of TDG and APE1 [122]. This study reported that RNF4 works as a regulator of DNA methylation by enhancing the enzymatic activities of TDG and APE1 in DNA demethylation.

The CRL4 E3 ubiquitin ligase has been shown to promote ubiquitination and proteasomal degradation of TDG [123]. TDG ubiquitination by CRL4 is dependent on the interaction of TDG with PCNA. The PCNA-interacting peptide (PIP) motif of TDG interacts with the PCNA PIP motif. TDG is also shown to interact with SUMO1 [124,125]. This interaction leads to the conjugation of TDG with SUMO1, which reduces the AP-site binding affinity of TDG, resulting in increased enzymatic turnover. SUMO1 covalently binds to the N-terminal regulatory domain of TDG and competes with its DNA-binding activity [126].

### 4.4. MPG/AAG

MPG has been shown to interact with the estrogen receptor α (ERα) [127]. ERα increases MPG acetylation and stabilizes the binding of MPG to hypoxanthine-containing oligos [128]. It was suggested that DNA-bound ERα recruits MPG to the chromatin to facilitate DNA repair. MPG also interacts with PCNA [129], suggesting that PCNA plays a role in the recruitment of MPG to the damaged site. Another study demonstrated that MPG interacts with both homologues of the RAD23 proteins (hHR23) [130]. The results indicated that MPG forms a complex with hHR23, and this complex has a greater binding affinity for damaged DNA. The binding of MPG to hHR23 stimulates the glycosylase activity. It was also demonstrated that both the N-terminal and C-terminal regions of MPG are critical for its interaction with hHR23.

A recent study indicated that UV-damaged DNA-binding protein (UV-DDB) interacts with MPG [131]. The findings indicate that UV-DDB facilitates the repair of N^6^-ethenoadenine (εA) and hypoxanthine (Hx) lesions by MPG. Using gel mobility shift assays, it was demonstrated that UV-DDB recognizes εA and Hx lesions four-to-five times better than non-damaged DNA. This study also demonstrated that UV-DDB stimulates MPG activity by 4–5-fold. MPG also interacts with ubiquitin-like PHD and RING finger domains 1 (UHRF1) [132]. The functional consequences of these interactions are not known.

### 4.5. MUTYH

MUTYH interacts with PCNA and RPA [133]. PCNA binds to the C terminus of MUTYH, and RPA binds to the N terminus of MUTYH. It is suggested that the interaction of MUTHY with PCNA and RPA indicates that MUTYH functions in long patch BER (LP-BER), specifically in the removal of adenine opposite 8-oxoG during DNA replication [134,135]. MUTYH has been shown to interact with the mismatch repair protein hMSH6 [136]. It was reported that this interaction stimulates the DNA binding and glycosylase activity of MUTYH. MUTYH also interacts with SIRT6 [137]. SIRT6 is suggested to enhance the activities of MUTYH. Specifically, the mono-ADP-ribosyltransferase activity of SIRT6 has been shown to be important for the stimulation of MUTYH activity. The E3 ubiquitin ligase Mule physically interacts with and ubiquitinates MUTYH [138]. Mule may regulate MUTYH protein levels.

### 4.6. NTHL1

NTHL1 interacts with PCNA, p53, and XPG [139]. Using a pull-down assay, it was demonstrated that NTHL1 physically interacts with these proteins. Evidence was also provided to show that p53 and XPG stimulate the DNA glycosylase/AP lyase activity of NTHL1. Other studies reported that XPG enhances the binding of NTHL1 to the thymine glycol-containing DNA, which stimulates the enzymatic activity of NTHL1 [140,141]. NTHL1 overexpression has been shown to sequester XPG, resulting in replication stress and accumulation of double-stranded breaks (DSBs) [142]. Further, overexpression of NTHL1 results in increased cytotoxicity and UV light sensitivity. It was shown that an N-terminal mutant of NTHL1 lacking the nuclear localization signal was still able to interact with XPG, indicating that another region of NTHL1 is responsible for interacting with XPG [143].

### 4.7. OGG1

OGG1 has been shown to interact with protein kinase C (PKC) [144]. PKC phosphorylates OGG1, and it was reported that phosphorylation does not alter the enzymatic function of OGG1. It was shown that chromatin-associated OGG1 is phosphorylated, while nuclear matrix-associated OGG1 is unphosphorylated. This suggests that phosphorylation may direct the compartmentalization of OGG1 in the nucleus. OGG1 has been shown to interact with the Rad9-Rad1-Hus1 (9-1-1) checkpoint protein complex [145]. It was demonstrated that OGG1 directly interacted with all three subunits of the 9-1-1 complex, and this interaction enhances the DNA binding and glycosylase activity of OGG1.

The telomere-associated complex, CTC1-STN1-TEN1 (CST), has been shown to interact with OGG1 [146]. Using a proximity ligation assay (PLA) and Co-IP, the STN1 subunit was identified as interacting with OGG1 in both H_2_O_2_-treated and untreated cells. Co-IP results indicated that OGG1 interacts weakly with CTC1 when cells are treated with H_2_O_2_, but not with the TEN1 subunit. Further, this study demonstrated that the CST complex enhances OGG1 activity by 5.9-fold on an 8-oxo-G-containing substrate.

The Cut homeobox CUX1 and CUX2 proteins have been shown to interact with OGG1 [147,148]. The Co-IP results demonstrated that CUX1 and CUX2 form a complex with OGG1 [147]. The Cut repeat domains of CUX1 and CUX2 proteins have been shown to stimulate the glycosylase and AP-lyase activities of OGG1 by enhancing the binding of OGG1 to 8-oxoG-containing DNA substrates [147,148,149]. Using sodium borohydride trapping of OGG1, it has been demonstrated that Cut repeats stimulate the formation of a Schiff base intermediate and the binding of OGG1 to the 8-oxoG DNA substrate [147].

OGG1 repairs 8-oxoG DNA lesions via both the SN-BER and LP-BER pathways [182,183,184]. Studies indicate that 8-oxoG lesions in non-replicating DNA are repaired through SN-BER, while 8-oxoG lesions arising or detected during DNA replication are repaired through LP-BER. OGG1 has also been shown to directly interact with chromodomain helicase DNA-binding protein 4 (CHD4), a component of the nucleosome remodeling and histone deacetylation (NuRD) complex [150]. Oxidative damage induces the interaction between CHD4 and OGG1. CHD4 is an essential part of the DNA damage response (DDR) and accumulates at the sites of DNA damage [151]. CHD4 plays a role in chromatin relaxation at the DNA damage site, and its recruitment to this site is PARP1-dependent [152].

The E3 ubiquitin ligase NEDD4L ubiquitinates OGG1 and modulates the levels of OGG1 [153]. This regulation is crucial for maintaining the balance of OGG1 levels and the BER capacity during oxidative stress.

### 4.8. NEILs

NEIL1 interacts with the Werner syndrome protein (WRN) [154]. WRN associates with the C-terminus of NEIL1 and facilitates the early damage-sensing step of BER. Oxidative stress enhances the NEIL1-WRN association. WRN stimulates NEIL1 activity, and in turn, NEIL1 inhibits the helicase activity of WRN. NEIL1 interacts with PCNA via its C-terminus [155]. PCNA stimulates NEIL1 activity by enhancing the loading of NEIL1 onto the DNA substrate. Flap endonuclease 1 (Fen-1) has also been shown to stimulate NEIL1 activity by interaction with its C-terminus [156]. Interestingly, Fen-1 and NEIL1 colocalize in the nucleus. NEIL1 interacts with the 9-1-1 complex [157], and this interaction significantly stimulates the NEIL1 DNA glycosylase activity. RPA has been shown to interact with the CID of NEIL1 and plays a role in regulating the repair process during DNA replication [158,159]. The interaction of NEIL1 with PCNA, RPA, Fen-1, and the 9-1-1 complex indicates that NEIL1 repairs oxidative lesions via both the SN-BER and LP-BER pathways [156]. NEIL1 has been shown to interact with the mitochondrial single-strand DNA-binding protein (MtSSB) [160]. This interaction is important for maintaining mitochondrial DNA integrity.

NEIL2 has been shown to interact with the transcriptional coactivator p300 [161]. The p300 coactivator regulates NEIL2 activity by acetylation, which significantly decreases its activity. Another study demonstrated that PKC phosphorylates NEIL2 and modulates its activity [162].

NEIL3 plays a major role in psoralen-interstrand cross-link (ICL) repair [185]. NEIL3 has been shown to interact with the RUVBL1/2 complex, which is associated with the Fanconi anemia/BRCA pathway [163]. Using a pulldown assay, it has been demonstrated that NEIL3 primarily binds to RUVBL2. NEIL3 also interacts with TRAIP, a master regulator of psoralen-ICL repair [164]. Specifically, TRAIP-dependent Cdc45-MCM-GINS (CMG) ubiquitylation recruits NEIL3 to the psoralen-ICL repair site. Another study demonstrated that NEIL3 directly interacts with the zinc finger protein 212 (ZNF212) [165], which is a binding partner of TRAIP and promotes NEIL3 recruitment to the ICL lesions.

### 4.9. XRCC1

The checkpoint kinase 2 (Chk2) has been shown to interact with and form a complex with XRCC1 [166]. It was reported that Chk2 phosphorylates the T284 residue on XRCC1, and the phosphorylated form of XRCC1 exhibited increased interaction with DNA glycosylases.

The ADP ribosylation factor-like GTPase 6 interacting protein 5 (ARL6IP5 or JWA) has been shown to interact with XRRC1 and colocalize with XRCC1 foci at sites of oxidative DNA damage [167]. JWA appears to protect XRCC1 from ubiquitination and proteasomal degradation. The UHRF2 ubiquitin E3 ligase has been shown to physically interact with the BER complex [168]. This study reported that UHRF2 catalyzes K33-linked polyubiquitination of XRCC1, and that polyubiquitination stimulates XRCC1 interaction with RAD23B, which facilitates the loading of TDG onto the BER complex. XRCC1 is also regulated by SUMOylation [186]. This study reported that the alkylating agent methyl methanesulfonate (MMS)-induced PARylation regulates the SUMOylation of XRCC1.

### 4.10. APE1

SIRT6 interacts with APE1 and enhances its endonuclease activity [137]. The mono-ADP-ribosyl transferase activity of SIRT6 is important for the stimulation of APE1. RNF4 has been shown to interact with and enhance the enzymatic activity of APE1 [122]. This study demonstrated that APE1 and TDG interact with the N-terminal region of RNF4. RNF4 is a regulator of DNA demethylation and works by enhancing the enzymatic activities of APE1 in DNA demethylation. It was suggested that RNF4 regulates demethylation through an APE1 and TDG interaction. The RNF4 interaction with APE1 and TDG is required for active demethylation. Another study has shown that APE1 stimulates the turnover of TDG [80]. RNF4 may act as a molecular scaffold to bring together TDG and APE1 for efficient BER repair [122]. APE1 interacts with flap endonuclease 1 (FEN1) and PCNA. This interaction influences the rate of LP- BER [170]. APE1 also interacts with Pol β [187]. When Pol β is unable to remove the dRP from the 3′ end and SN-BER is stalled, the interaction of APE1 with FEN1 and PCNA assists in the recruitment of components needed for the LP-BER.

The p300 acetyltransferase interacts with and acetylates APE1 at Lys6 and Lys7 residues in its N-terminal domain [171]. It was reported that there is no difference in the AP-endonuclease activity of acetylated and unmodified APE1. Specifically, it was suggested that acetylation provides a regulatory switch for different functions of APE1, including repair of AP sites and SSBs, activation of transcription factors, and Ca^2+^ dependent regulation of parathyroid hormone expression. Genotoxic insults such as exposure to H_2_O_2_ and MMS result in increased APE1 acetylation [68]. It was demonstrated that the SIRT1 protein deacetylase antagonizes APE1 acetylation by deacetylating at Lys6 and Lys7 of APE1. This study suggested that genotoxic insults upregulate SIRT1, which acts as a feedback mechanism for the regulation of APE1 acetylation. SIRT1 also promotes the interaction of APE1 and XRCC1, which stimulates the AP endonuclease activity of APE1 [68]. The E3 ubiquitin ligase MDM2 has been shown to interact with the C-terminus of APE1 [172]. MDM2 ubiquitinates the Lys 24, Lys25, and Lys27 residues near the N-terminal of APE1. It was shown that the ubiquitination of APE1 by MDM2 is increased in the presence of ataxia-telangiectasia mutated (ATM).

The CST complex has also been shown to interact with APE1 [146]. Co-IP results from this study indicated that APE1 interacts with the CTC1 and STN1 subunits when cells were treated with H_2_O_2,_ but the TEN1 subunit does not interact with APE1. APE1 cleavage activity results from this study revealed that, in the presence of the CTC1 subunit alone, APE1 endonuclease activity was enhanced by 2.7-fold. In the presence of CTC1, in combination with either STN1 or TEN1, APE1 endonuclease activity was enhanced by 1.2-fold. Finally, in the presence of the CST complex, APE1 endonuclease activity was enhanced by 2-fold. APE1 endonuclease activity was also shown to be stimulated by the CUT domains of the CUX1 protein [169]. The interaction of CUX1 and APE1 has been demonstrated by Co-IP and pull-down assays.

### 4.11. PNKP

DNA-dependent protein kinase (DNA-PK) and ATM have been shown to phosphorylate and regulate PNKP [173]. It was reported that both DNA-PK and ATM phosphorylate PNKP at the S114 and S126 amino acid residues, and that inactivation of these kinases individually or together resulted in reduced PNPK at the DNA damage site. Further, it was observed that PNKP protein harboring mutations at S114 and S126 had reduced affinity for DNA.

### 4.12. DNA Pol β

The 9-1-1 complex physically interacts with and stimulates Pol β activity [174]. The 9-1-1 complex is a DNA damage sensor that is recruited to DNA damage sites. It was shown that the 9-1-1 complex stimulates Pol β by enhancing the DNA strand displacement synthesis. The authors suggested that the 9-1-1 complex plays a role in the recruitment of Pol β to the DNA damage repair site. WRN was also shown to interact with and stimulate Pol β activity [175]. The helicase activity of WRN stimulates Pol β DNA strand displacement synthesis. Another study by the same group reported that the exonuclease activity of WRN can cooperate with the polymerase activity of Pol β to remove misincorporated bases [176]. The authors suggested that WRN and Pol β together play a role in LP-BER. WRN was also shown to recruit the chromatin assembly factor 1 (CAF-1) to the DNA damage site [188].

The N-terminal dRP-lyase domain of Pol β interacts physically with p300 [178]. The K72 residue of Pol β is important for the dRP-lyase activity. p300 regulates Pol β by acetylating K72, and this acetylation results in the inhibition of the dRP-lyase activity of Pol β. The E3 Ubiquitin ligases Mule and ARF have been shown to regulate Pol β by ubiquitination [179]. Pol β is ubiquitinated on the Lys41, Lys61, and Lys81 residues. This study suggested that Mule and ARF control the steady-state levels of Pol β by ubiquitination and modulate BER.

The CST complex, in addition to OGG1 and APE1, also interacts with Pol β [146]. PLA results indicated that there is an interaction between STN1 and Pol β. Under oxidative stress, the interaction between STN1 and Pol β is increased by 2-fold. Co-IP results revealed that CTC1 interacts weakly with Pol β only in cells treated with H_2_O_2_. Both the STN1 and TEN1 subunits interact strongly with Pol β when cells are treated with H_2_O_2_, but they only interact weakly in untreated cells. Pol β gap-filling activity results revealed that, in the presence of CTC1 or the STN1 subunit alone, or the entire CST complex, activity was enhanced. This study also demonstrated that CTC1, STN1, and the entire CST complex enhance the strand displacement and lyase activity of Pol β.

The CUT domains of CUX1 were shown to stimulate the polymerase, dRP-lyase, and strand-displacement activities of Pol β [177]. The Co-IP results indicated that CUX1 interacts with Pol β. This study also reported that the CUT domains stimulate the bypass of intrastrand G-crosslink by Pol β. For detailed information about the CUT domains, please read a recently published review on CUT domain proteins [189].

### 4.13. LigIIIα

The LigIIIα-XRCC1 complex interacts with the hMre11/hRAD50/NBS1 (MRN) complex and plays a role in the alternative pathway of nonhomologous end joining (alt-NHEJ)9 [180]. In this study, the authors reported that cells that are defective in the classic-NHEJ (C-NHEJ) pathway have elevated levels of LigIIIα, but the amount of LigIIIα associated with MRN was lower than in wild-type cells. However, following DNA damage, there was a dramatic increase in the association of LigIIIα with MRN in C-NHEJ defective cells. Using a pulldown assay, the study demonstrated that MRN subunits directly interacted with the LigIIIα-XRCC1 complex. In mitochondrial protein extracts, LigIIIα has been shown to interact with DNA polymerase γ (Pol γ) using co-immunoprecipitation [181]. The C-terminus of Pol γ interacts with the central domain of LigIIIα.

### 4.14. APTX

APTX has been shown to interact with p53 [102]. Co-IP results from this study revealed that cells treated with H_2_O_2_ show increased interaction between p53 and APTX. They also demonstrated that p53 interacts with the FHA and HIT domains of APTX. This study also revealed that nucleolin interacts with the FHA domain of APTX; they both co-localize at the nucleoli.

## 5. Conclusions

In summary, the SN-BER pathway is a highly coordinated process involving a dynamic network of enzymes working together to repair DNA base lesions. Key to this process is the interaction between SN-BER components that ensures the efficient and regulated execution of repair. Two models have been proposed to explain the coordination of the SN-BER process for efficient repair of base lesions: “Passing the Baton” and “BERosome Repair Complex”. Evidence supporting the “Passing the Baton” includes the findings that BER enzymes tightly bind to their products, which helps sequester cytotoxic intermediate products and efficiently pass them to the next enzyme in the pathway [76,90,190]. Additionally, APE1 has been shown to stimulate turnover of DNA glycosylases, such as MUTYH, TDG, and OGG1, by accelerating the rate-limiting product release step [76,77,78,79,80,81,82,83,84]. On the other hand, the “BERosome Repair Complex” model is supported by findings that XRCC1 interacts with DNA glycosylases, such as UNG2, OGG1, NTHL1, MPG, NEIL1, and NEIL2 [58,59,60,61]. Additionally, XRCC1 interacts with APE1 [89], Pol β [66], and LigIIIα [99]. Moor et al. characterized and detected multiple direct interactions between downstream components of the BER pathway, such as APE1-Pol β and APE1-PARP1 [96,106]. A recent study by Caglayan et al. demonstrated tight interactions between Pol β and APE1 with LigIIIα and XRCC1, providing evidence for the BER multiprotein complex consisting of XRCC1, Pol β, and LigIIIα [64]. However, it appears that no study has conclusively demonstrated a complete multiprotein complex comprising all the core components of the BER pathway. As XRCC1 has been shown to interact with all the core components of the SN-BER pathway [58,59,60,61,66,89,99], there might be a transient multiprotein complex with XRCC1 acting as a scaffold protein to form a repair complex at the DNA repair site. XRCC1 may provide a platform for transient interactions between the core components of the SN-BER pathway, resulting in the coordination of the SN-BER process. Further studies are needed to confirm the existence of a transient multiprotein complex at the BER repair site in vivo. As it is difficult to demonstrate transient multiprotein complexes using traditional protein–protein interaction studies, such as co-immunoprecipitation and pull-down assays, future studies might focus on demonstrating real-time interactions between BER core components and XRCC1 with a combination of in vivo and in vitro studies.

The activity of upstream BER enzymes must be regulated and coordinated with the levels and activities of the downstream enzymes in the pathway. If the enzyme activity is not regulated, it could lead to the accumulation of cytotoxic BER intermediates. The activity of BER enzymes appears to be mainly regulated by PTMs, such as acetylation (APE1, NEIL2) [161,171], methylation (Pol β) [191], phosphorylation (PNKP, OGG1, NEIL2, XRCC1) [102,103,144,162,166,173], SUMOylation (MBD4, TDG, XRCC1) [117,124,125,126,186], and ubiquitination (UNG2, TDG, MUTYH, OGG1, NEIL3, XRCC1, APE1, Pol β) [109,123,138,153,164,168,172,179], which could impact the nature of protein–protein interactions. Phosphorylation, methylation, and acetylation of BER proteins result in altering the enzymatic activity, while SUMOylation and ubiquitination result in the degradation of BER enzymes. The interaction between DNA glycosylases, such as OGG1, NEIL1, and TDG, with PARP1 suggests that PARP1 plays a major role in modulating the activity of DNA glycosylases. XRCC1 is another critical scaffold protein that interacts with multiple DNA glycosylases, including UNG2, OGG1, NTHL1, MPG, NEIL1, and NEIL2. Further, APE1 has been shown to stimulate the turnover of DNA glycosylases, such as TDG, MUTYH, and OGG1. Additionally, Pol β interacts with several DNA glycosylases, including AAG, OGG1, NTHL1, MBD4, UNG2, and SMUG1. NEIL1 also interacts with PNKP, XRCC1, Pol β, and LigIIIα. Further studies on the interaction of PARP1, XRCC1, APE1, and Pol β with other DNA glycosylases are needed to better understand how the DNA glycosylases are regulated in the SN-BER process. High-throughput protein interactome networks of SN-BER components, such as those provided by BioGRID, suggest a complex web of interactions. However, confirmatory studies using more focused, low-throughput experimental approaches are required to provide direct evidence for these interactions and additional mechanistic insights. Such studies will enhance our understanding of how SN-BER components are regulated, not only within the context of BER but also in relation to other DNA repair mechanisms and cell-signaling pathways.

## Figures and Tables

**Table 1:** Interaction of SN-BER components with other proteins.

BER Component	Proteins That Interact	Functional Role of Interactions	References
UNG2	RPA2, PCNA	Recruits UNG2 to the replication fork to scan for uracil	[107,108]
	CRL4 E3, Vpr	Regulates UNG2 protein levels	[109,110,111,112]
SMUG1	CRL4 E3, Vpr	Regulates SMUG1 protein levels	[109,110,111,112]
MBD4	MLH1, FADD, HDAC, SIN3a, RPF	Regulates the apoptotic response to diverse DNA lesions	[23,113,114,115]
	UHRF1 E3 ligase, SUMO1	Regulates MBD4 protein levels	[116,117]
TDG	CBP, p300, XPC-HR23B	Turnover of TDG	[118,119]
	DNMT3a, DNMT3b,	Recruits TDG to the mismatch repair site	[120,121]
	RNF4	Stimulates TDG activity	[122]
	CRL4 E3, PCNA, SUMO1	Regulates TDG protein levels	[123,124,125,126]
MPG/AAG	ERα, PCNA	Recruits MPG to the repair site	[127,128,129]
	hHR23, UV-DDB	Stimulates MPG activity	[130,131]
	UHRF1		[132]
MUTYH	PCNA, RPA		[133,134,135]
	hMSH6, SIRT6	Stimulates MUTYH activity	[136,137]
	E3 ubiquitin ligase mule	Regulates MUTYH protein levels	[138]
NTHL1	PCNA, p53, XPG	Stimulates NTHL1 activity	[139,140,141,142,143]
OGG1	PKC	Compartmentalization of OGG1 in the nucleus	[144]
	9-1-1 complex, CST, CUX1, CUX2	Stimulates OGG1 activity	[145,146,147,148,149]
	CHD4	Chromatin relaxation at the repair site	[150,151,152]
	E3 Ubiquitin ligase NEDD4L	Regulates OGG1 protein levels	[153]
NEIL1	WRN, PCNA, Fen-1, 9-1-1 complex	Stimulates NEIL1 activity	[154,155,156,157]
	RPA, MtSSB		[158,159,160]
NEIL2	p300, PKC	Regulates NEIL2 activity	[161,162]
NEIL3	RUVBL1/2 complex, TRAIP, ZNF212	Regulates NEIL3 recruitment to the ICL lesions	[163,164,165]
XRCC1	CK2	Regulates interaction between XRCC1 and APTX	[102,103]
	Chk2	Regulates interaction between XRCC1 and DNA glycosylase	[166]
	JWA, RAD23B E3 ubiquitin ligase UHRF2	Regulates XRCC1 protein levels	[167,168]
APE1	SIRT1, SIRT6, RNF4, CST, CUX1	Stimulates APE1 endonuclease activity	[68,122,137,146,169]
	FEN1, PCNA, p300		[170,171]
	E3 ubiquitin ligase MDM2	Regulates APE1 protein levels	[172]
PNKP	DNA-PK, ATM	Regulates PNKP	[173]
Pol β	9-1-1 complex, WRN, CST, CUX1,	Stimulate Pol β activity	[146,174,175,176,177]
	p300, E3 ubiquitin ligase Mule & ARF	Regulates Pol β	[178,179]
Ligase IIIα	Mre11:Rad50:Nbs1, Pol γ		[180,181]
APTX	p53, nucleolin		[102]

## Supplementary Materials

The following supporting information can be downloaded at: https://www.mdpi.com/article/10.3390/biom15060890/s1, Figure S1: UNG protein interactome; Figure S2: SMUG1 protein interactome; Figure S3: MBD4 protein interactome; Figure S4: TDG1 protein interactome; Figure S5: MPG protein interactome; Figure S6: MUTYH protein interactome; Figure S7: NEIL1 protein interactome; Figure S8: NEIL2 protein interactome; Figure S9: NEIL3 protein interactome; Figure S10: NTHL1 protein interactome; Figure S11: OGG1 protein interactomeOGG1 protein interactome; Figure S12: XRCC1 protein interactome; Figure S13: APE1 protein interactome; Figure S14: PNK protein interactome; Figure S15: Pol β protein interactome; Figure S16: LigIIIα protein interactome.
