# Supplementary material for: Protein–Protein Interactions in Base Excision Repair
Source: Biomolecules. 2025 Jun 18;15(6):890. doi: 10.3390/biom15060890 (PMC12190888; doi:10.3390/biom15060890)
Supplement: Supplementary file 1 [file biomolecules-15-00890-s001.zip › biomolecules-3660987-supplementary.pdf]

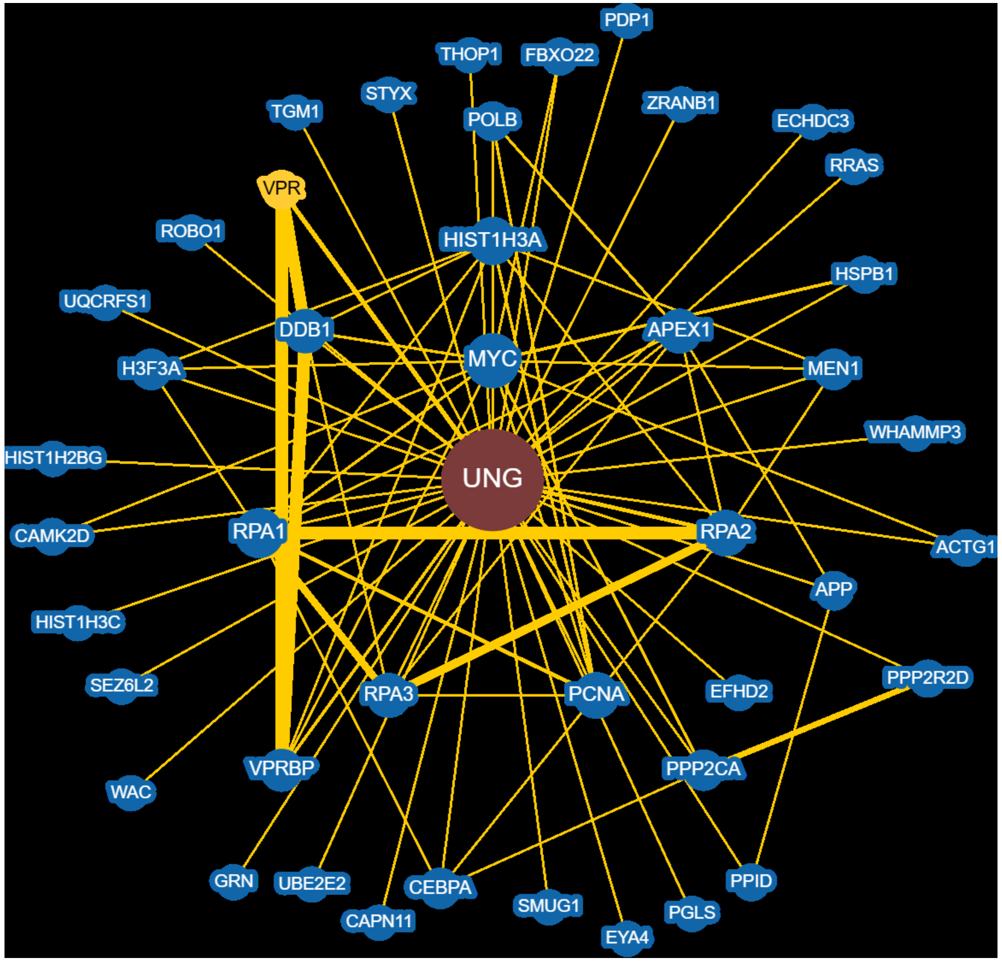

Figure S1. UNG protein interactome

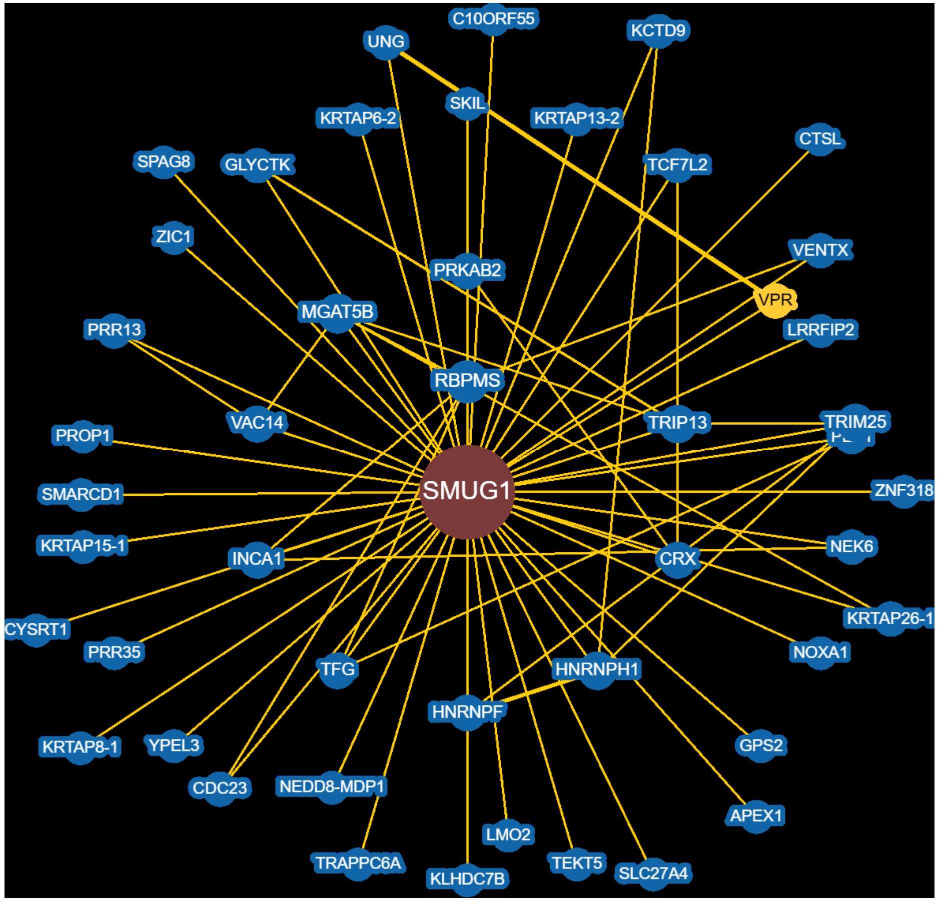

Figure S2. SMUG1 protein interactome

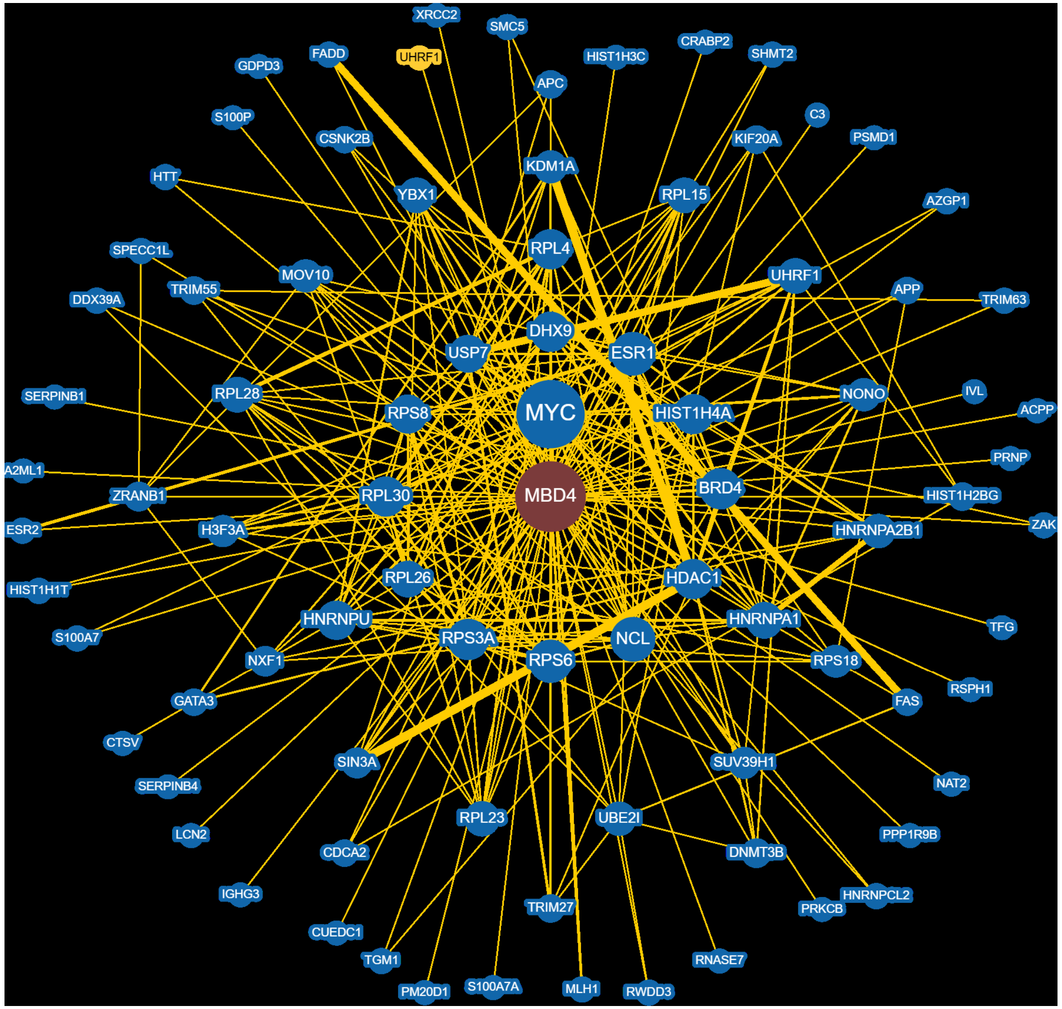

Figure S3. MBD4 protein interactome

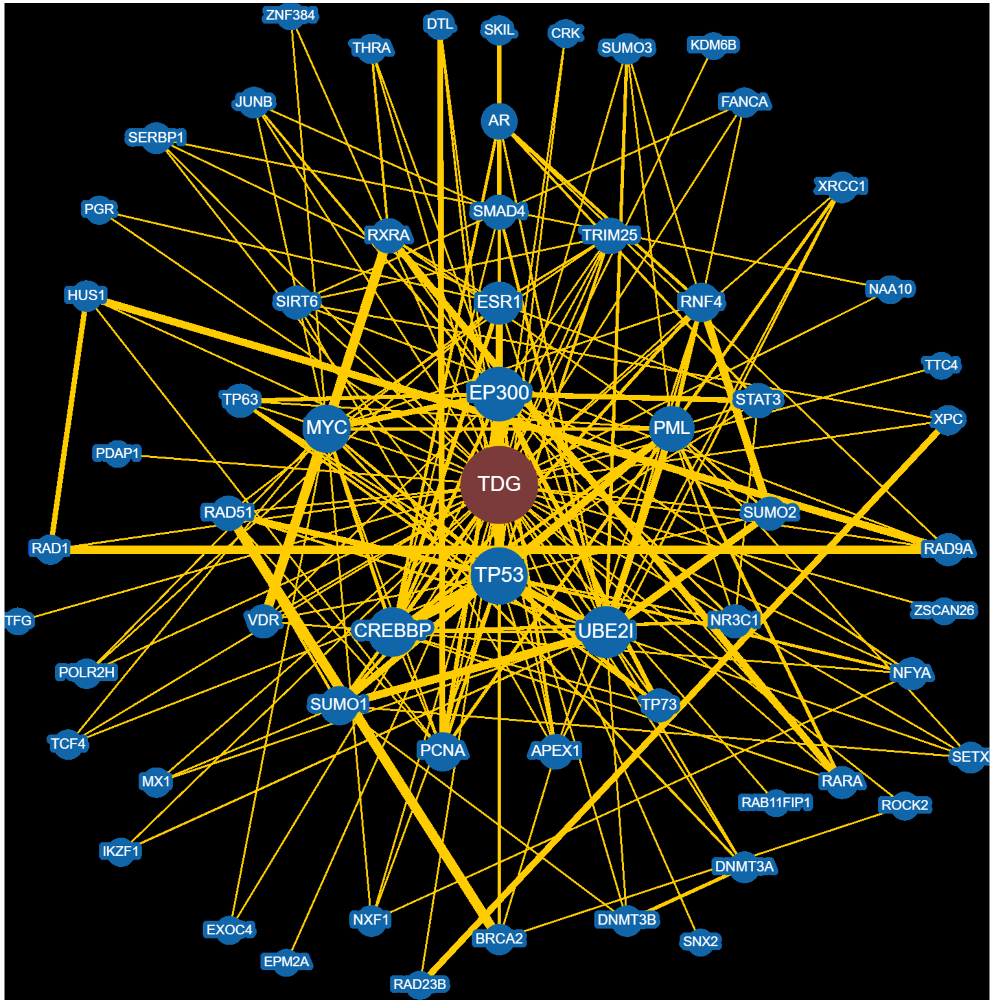

Figure S4. TDG1 protein interactome

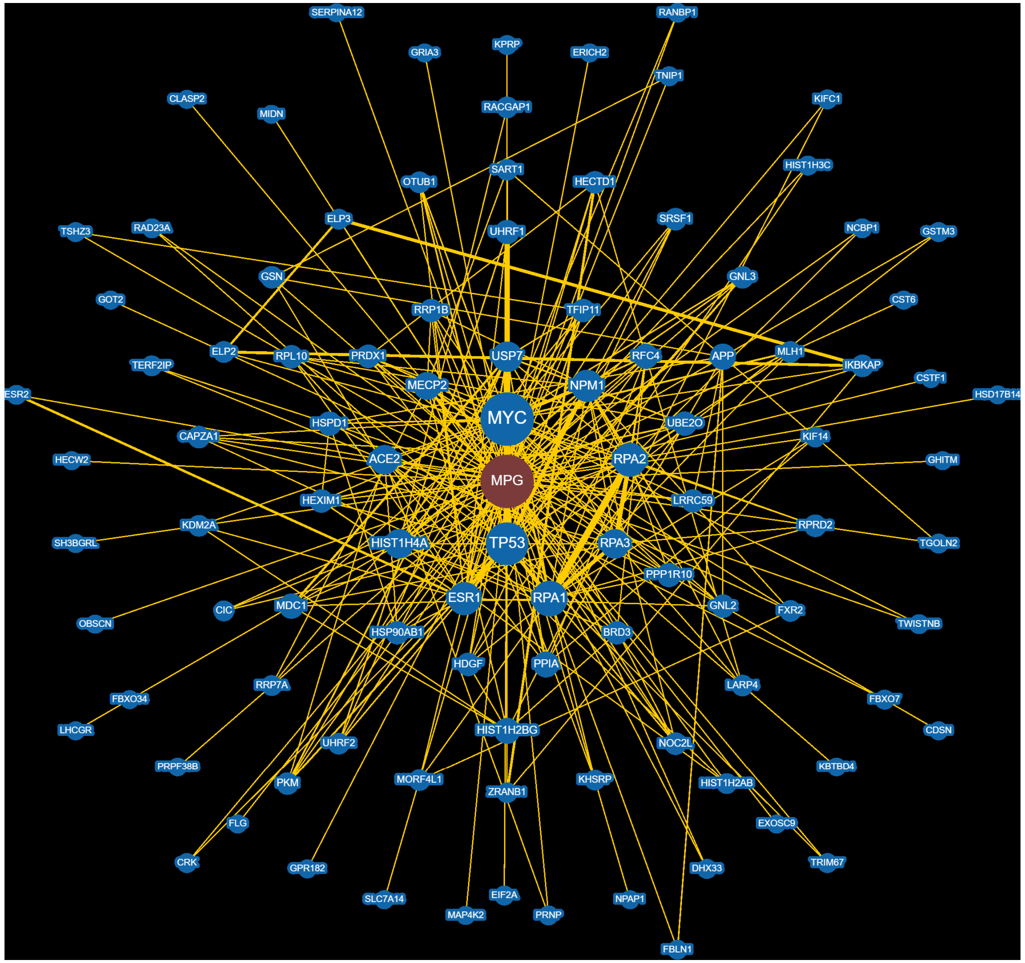

Figure S5. MPG protein interactome

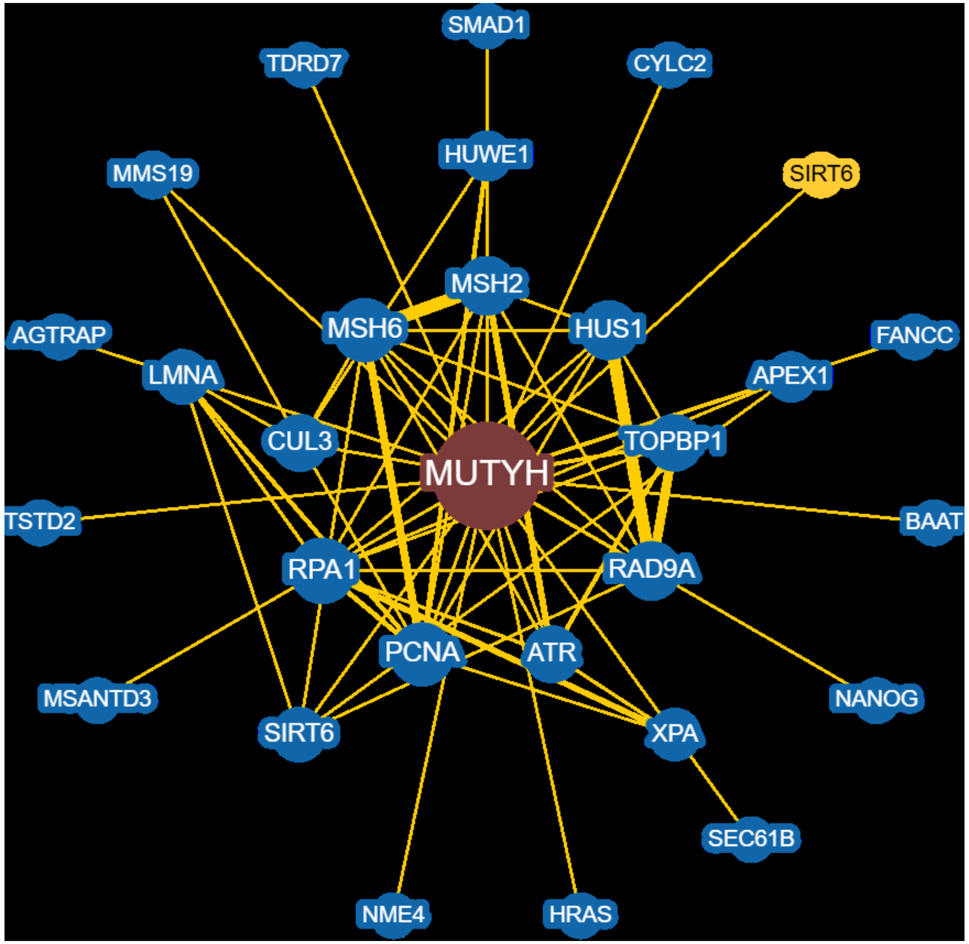

Figure S6. MUTYH protein interactome

NEIL1 protein interactome- BioGRID

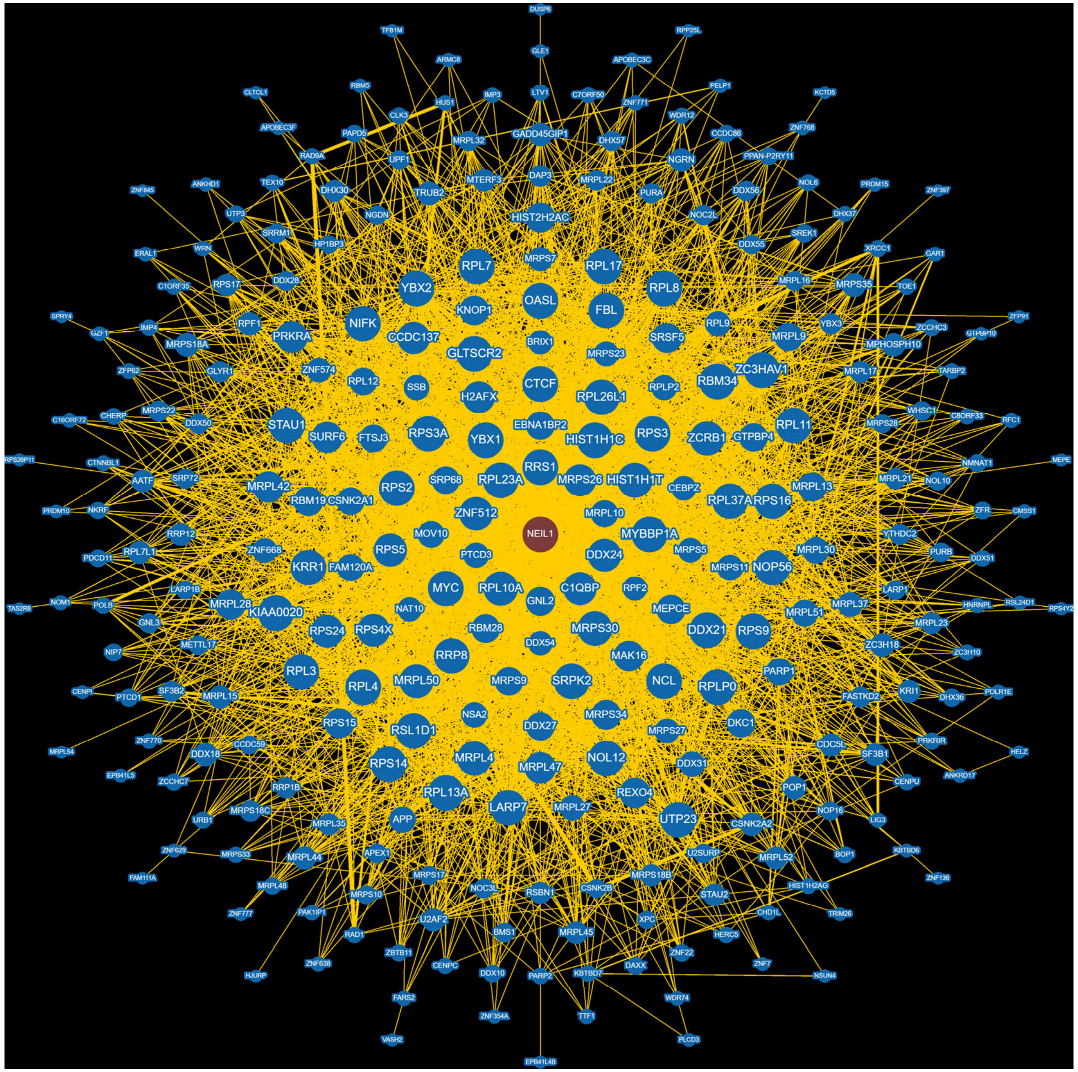

Figure S7. NEIL1 protein interactome

NEIL2 protein interactome- BioGRID

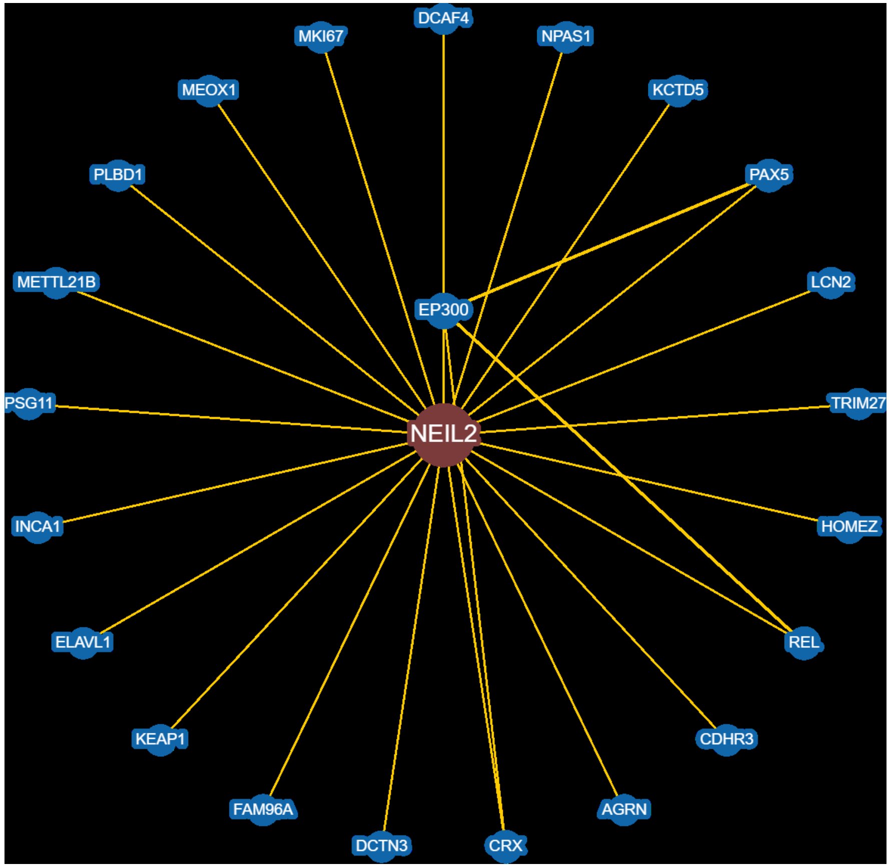

Figure S8. NEIL2 protein interactome

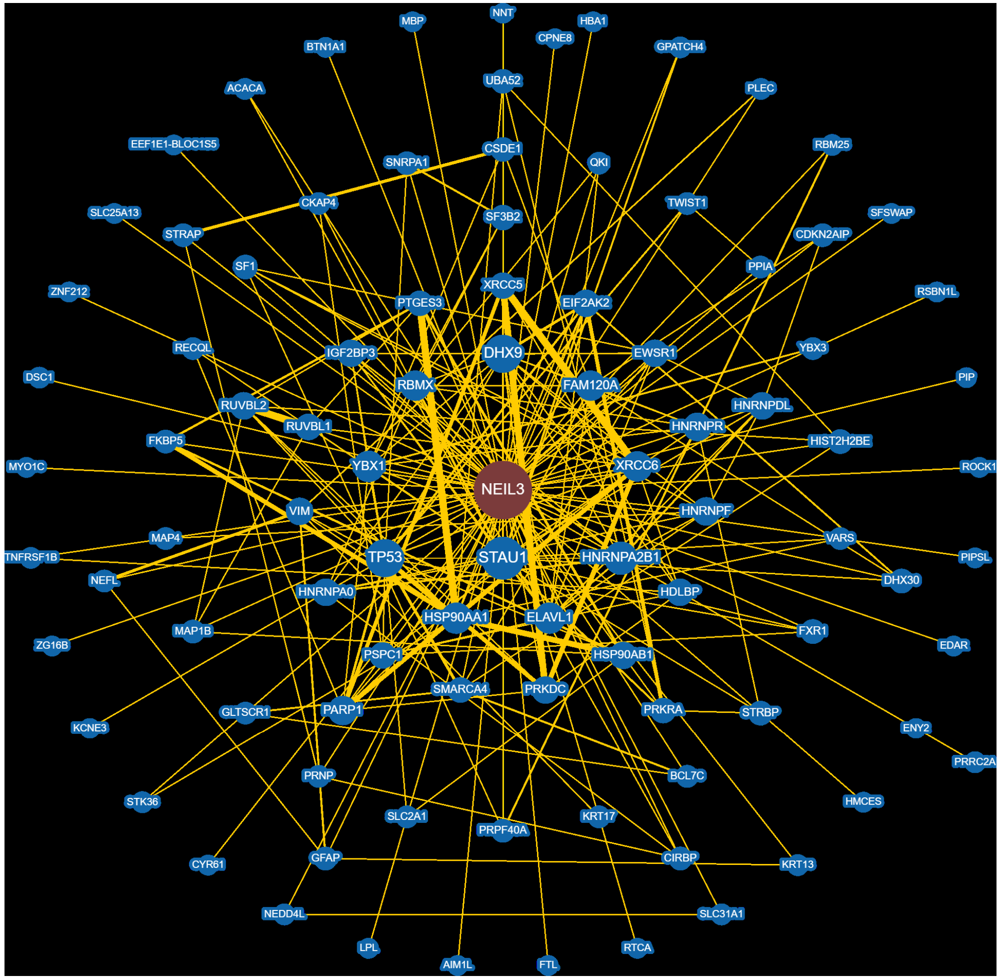

Figure S9. NEIL3 protein interactome

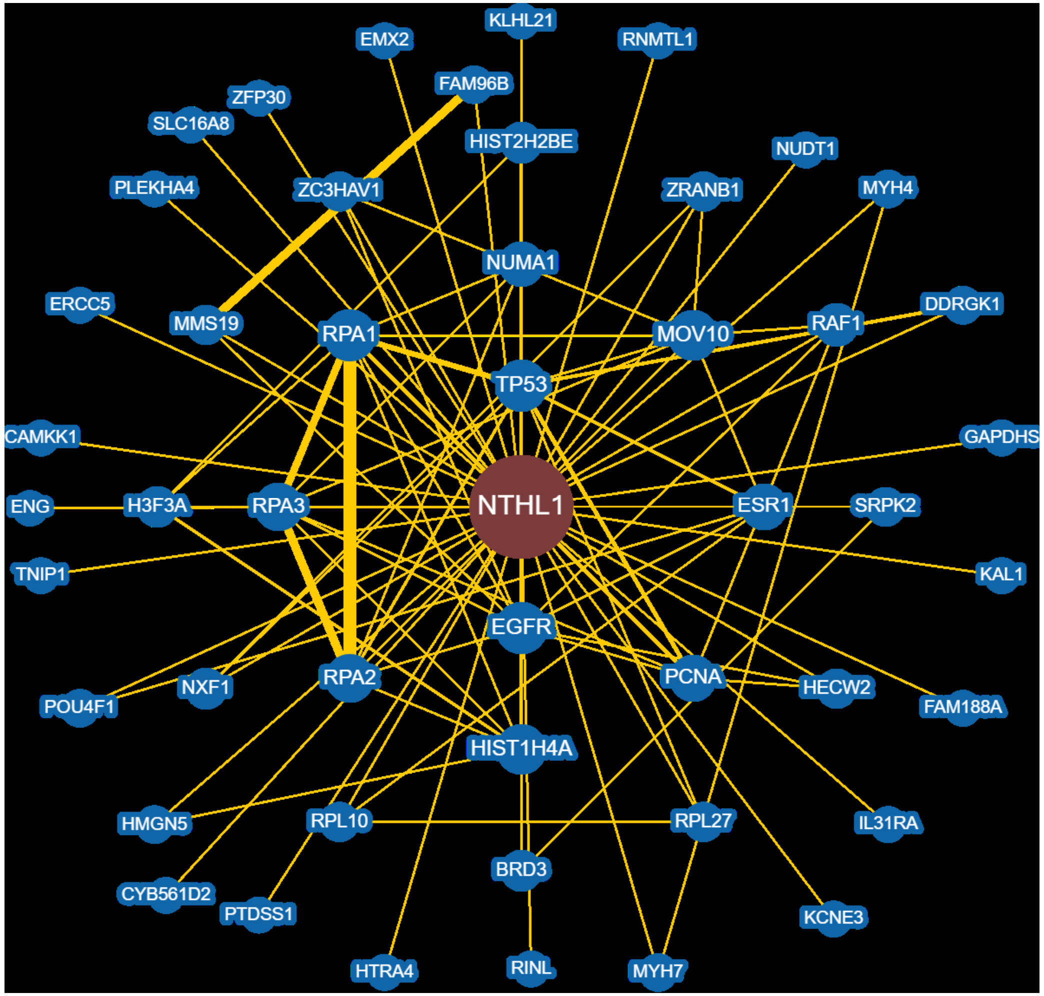

Figure S10. NTHL1 protein interactome

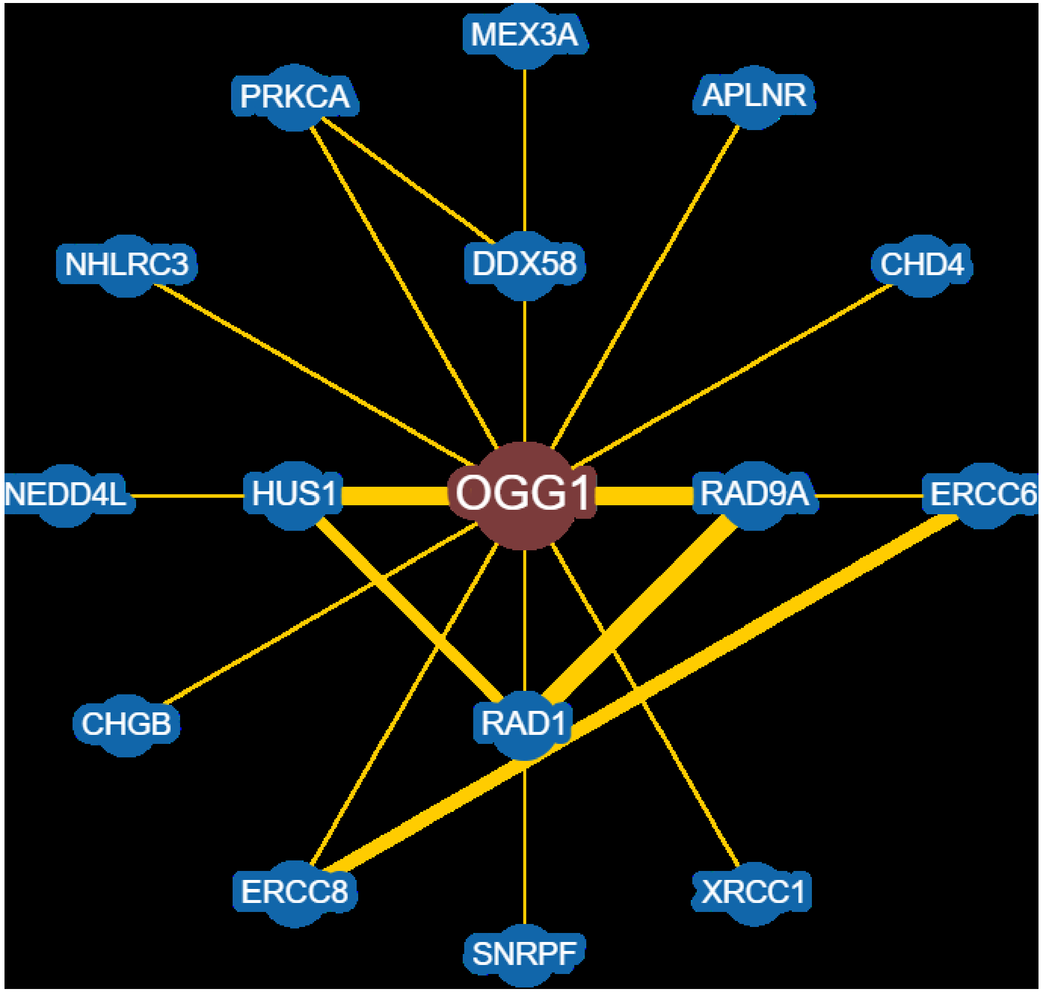

Figure S11. OGG1 protein interactome

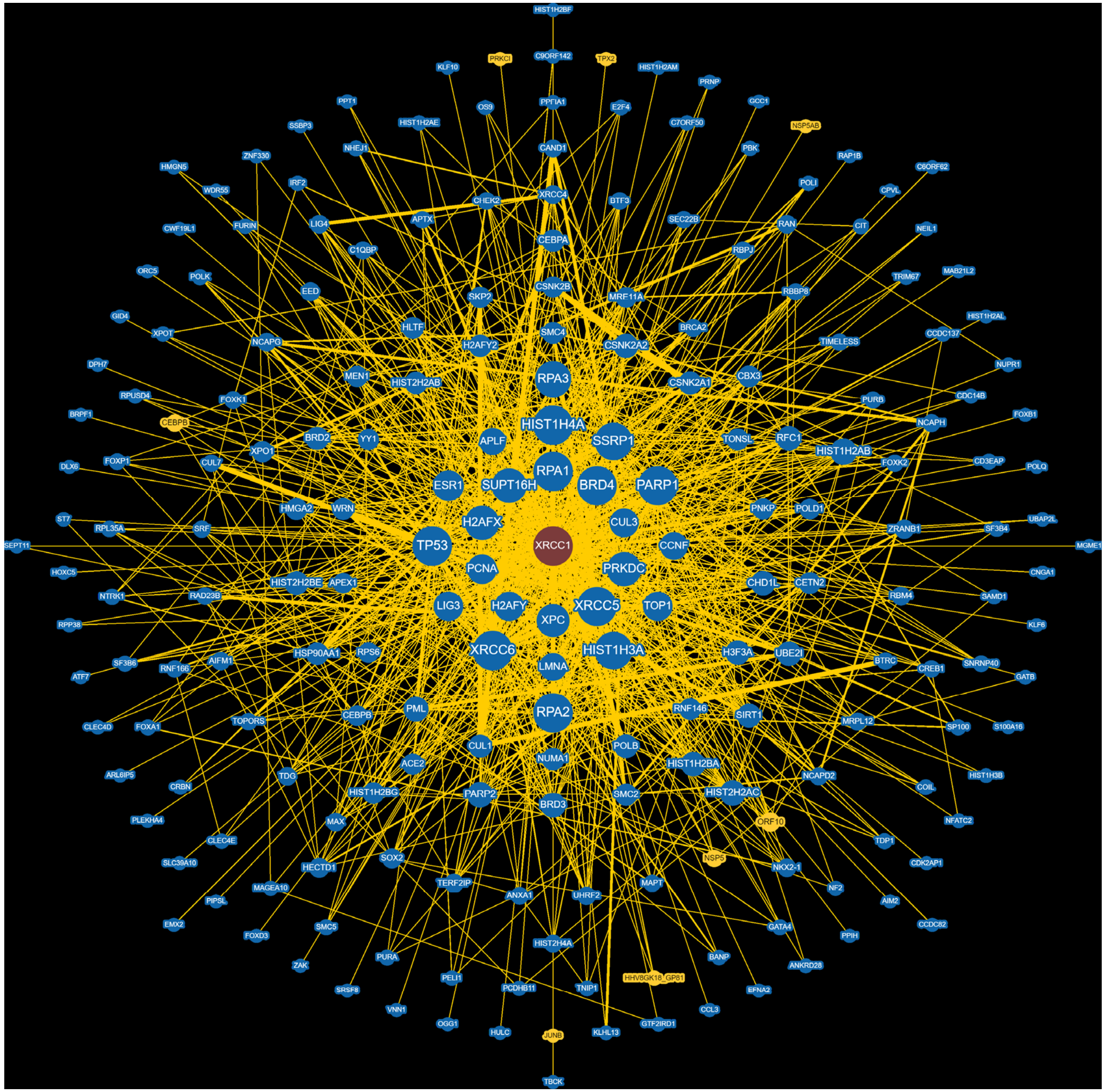

Figure S12. XRCC1 protein interactome

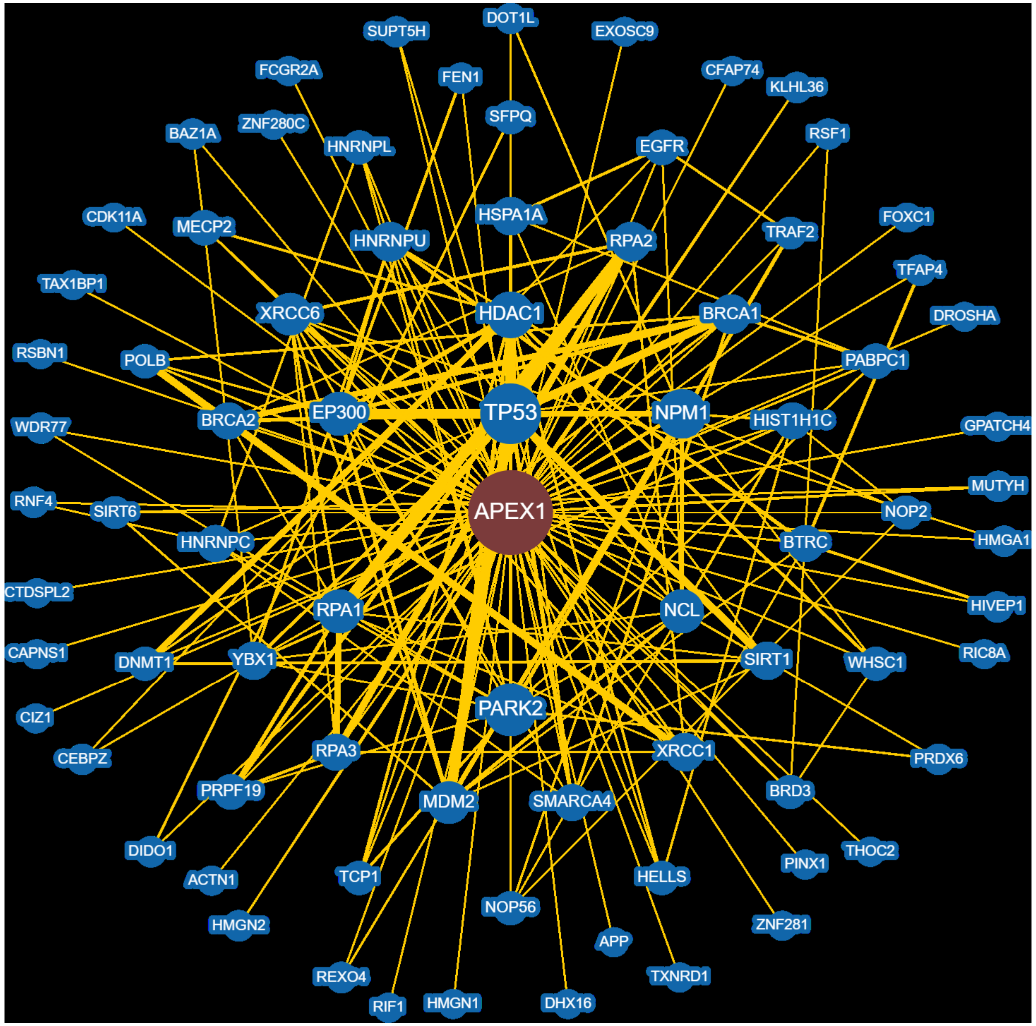

Figure S13. APE1 protein interactome

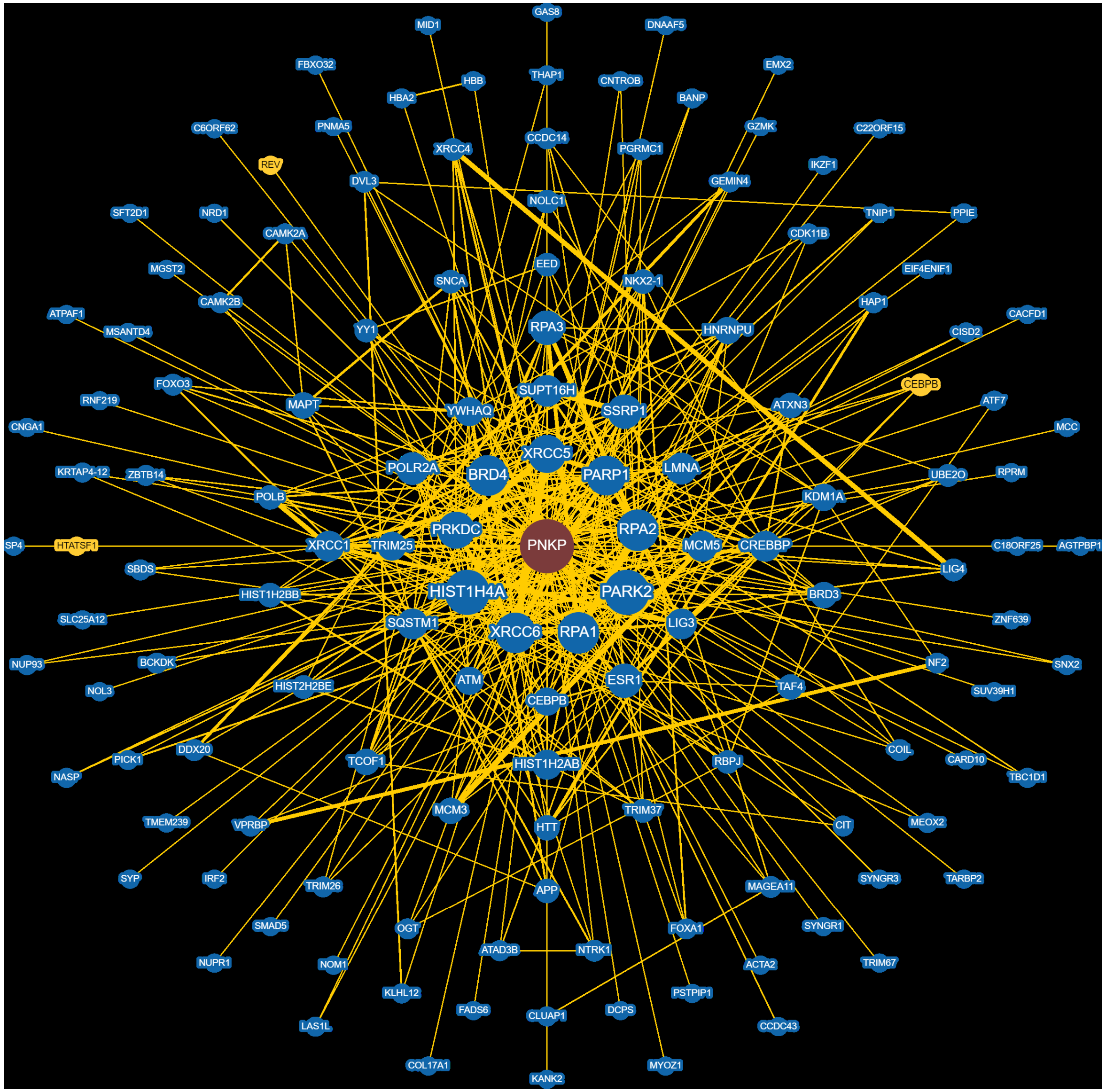

Figure S14. PNK protein interactome

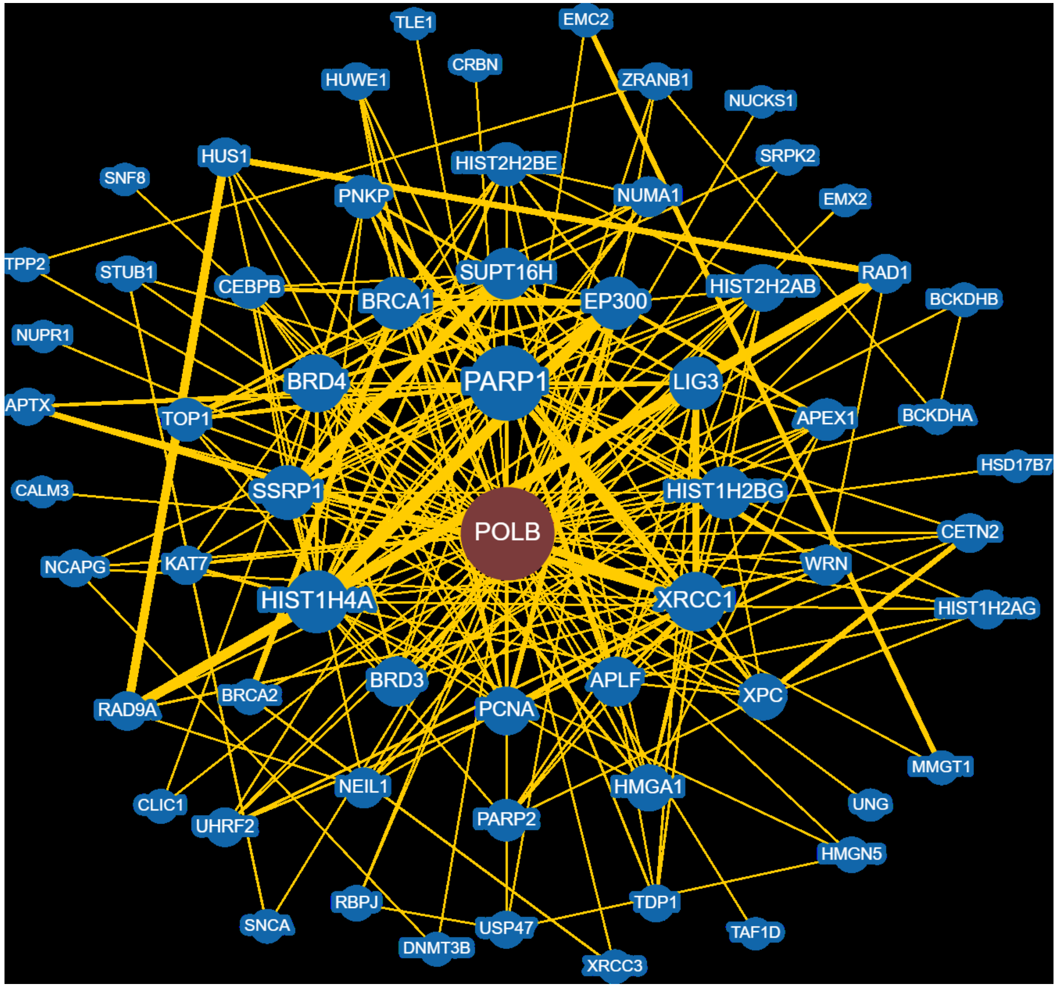

Figure S15. Pol β protein interactome

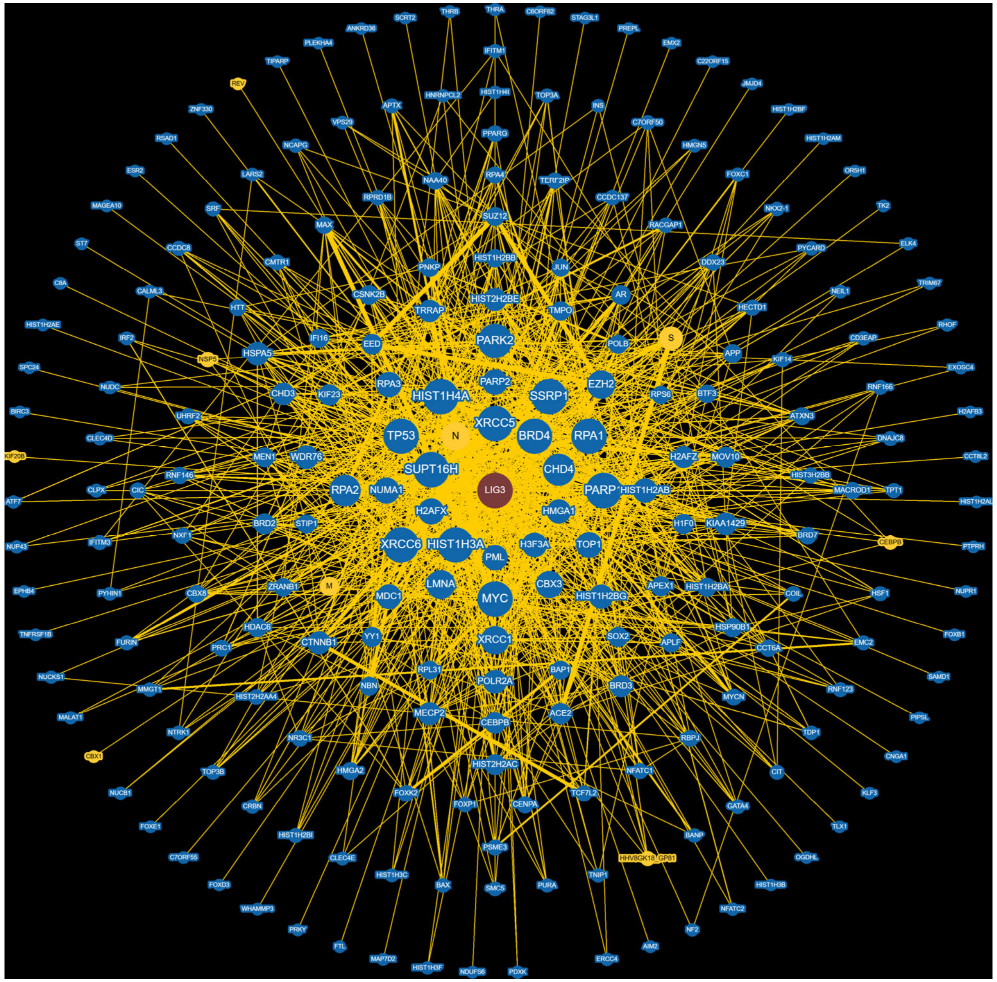

Figure S16. LigIIIα protein interactome
